# Effects of Whole-Body Vibration on Upper Extremity Function and Grip Strength in Patients with Subacute Stroke: A Randomised Single-Blind Controlled Trial

**DOI:** 10.1155/2019/5820952

**Published:** 2019-04-01

**Authors:** Jin-Young Ahn, Hyeongsu Kim, Chan-Bum Park

**Affiliations:** ^1^Department of Preventive Medicine, School of Medicine, Konkuk University, Seoul 05029, Republic of Korea; ^2^Department of Physical Therapy, Kyung Hee University Health Care System, Seoul 02447, Republic of Korea; ^3^Department of Physical Therapy, Yonsei University, Wonju 26493, Republic of Korea

## Abstract

**Background:**

Whole-body vibration has been used to improve motor function in chronic stroke patients, but its effect on patients with subacute strokes remains unclear.

**Objectives:**

We explored the effect of whole-body vibration on patients with subacute strokes.

**Methods:**

Participants were randomly allocated to a whole-body vibration (WBV) group (*n* = 30) or an upper- and lower-cycle (ULC) group (*n* = 30). Both groups received occupational therapy after these interventions. All participants received treatment for 30 min/day, 5 days/week, for 4 weeks. Both groups received the same conventional physical therapy.

**Results:**

The manual function test (MFT) score and grip strength improved after both WBV (*p* = 0.001 and *p* = 0.001, respectively) and ULC (*p* = 0.002 and *p* = 0.001, respectively), but the improvement was more pronounced (MFT *p* = 0.016; GS *p* = 0.023) after WBV.

**Conclusions:**

These findings suggest that the use of WBV and ULC was effective as remedial treatments for improving upper extremity motor function and increasing grip strength for patients with subacute strokes. The improvement was more pronounced for the WBV treatment. This trial is registered with KCT0003246.

## 1. Introduction

Stroke, which is a leading cause of long-term disability, is often associated with persistent involvement of an upper extremity [1]. The upper extremity function in stroke patients depends on several factors, including paresis severity, degree of spasticity, and extent of motor and sensory loss [2]. Upper extremity paresis after stroke is a leading cause of serious and long-term hand disability [3]. After a stroke, patients exhibit a complex pattern of upper extremity motor impairments resulting in the loss of functional abilities, such as grip and grasp [4], causing pain, joint contracture, and discomfort, which may lead to limb disuse and impede long-term functional recovery [5]. Additionally, because patients use the unaffected side more during arm action, it is necessary to apply therapy to the affected arm [6]. Of the various approaches used to improve motor function, the first is an effort to increase somatosensory input from the paretic hand using somatosensory stimulation to enhance the brain response [7].

Improving upper extremity motor function is important for increasing occupational engagement [8]. Use of vibration stimulation as an intervention has demonstrated improvement in affected upper extremity motor function for stroke patients since 1990 [8, 9].

Upper extremity motor recovery is aided by task-oriented practice [10]. Such training has been used to facilitate motor function [11]; various desired movements are learned, and inappropriate movements are reduced, thereby improving the adaptation of stroke patients [12]. Patients control strategy is improved by task-oriented training [13], which is more effective than traditional therapies [14].

Whole-body vibration (WBV) is a form of somatosensory stimulation used to rehabilitate stroke patients [15]. WBV affects proprioceptive systems [16]. Low-amplitude WBV (<20 Hz) induces muscular relaxation; medium-amplitude WBV (>50 Hz) triggers muscle soreness and haematoma [17]. WBV enhances muscle strength and power, affording neuromuscular adaptations similar to those produced by strengthening exercises [18].

Previous studies on chronic stroke patients showed that WBV improved walking speed, step length, stride length, double-limb support [19], and balance [20] and increased upper extremity function and strength but decreased upper extremity muscle tone [21]. However, although early rehabilitation of stroke patients is very important, no study has yet investigated the effects of WBV on upper extremity motor function and grip strength in subacute stroke patients; thus, we focused on this topic in this study.

## 2. Materials and Methods

### 2.1. Participants

We recruited patients with subacute stroke (*n* = 60) treated at a local rehabilitation centre in the Republic of Korea. The inclusion criteria were (1) that they experienced their first stroke within 6 months prior to recruitment, (2) a score ≥ 26 on the Mini-Mental Status Examination-Korean version, (3) an affected upper extremity score ≤ 2 on the modified Ashworth scale, (4) an affected upper extremity of Brunnstrom stage ≥ 3, (5) Manual Muscle Test (MMT) grade of >2/5 in the hemiparetic shoulder, and (6) a visual analogue pain scale score ≤ 4. The exclusion criteria were (1) another neurological disease, (2) any prestroke musculoskeletal abnormality, and (3) a score < 47 on the star cancellation test for visual spatial neglect. Written informed consent was obtained from all patients. The study was approved by the Institutional Review Board of Konkuk University (7001355-201802-HR-228).

A sample size of 30 of each group was calculated with an alpha of 0.05, power of 80%, an effect size of 0.7, and a drop-out rate of 10%, using the G-power program.

### 2.2. Procedures

This was a prospective two-group randomised controlled trial. Each of the 60 participants was randomly allocated to a WBV group (*n* = 30) or an upper- and lower-cycle (ULC) group (*n* = 30); each subject drew a card from a box containing two cards marked 1 (WBV group) or 2 (ULC group) without looking at the cards.

The WBV group received WBV (Galileo 2000, Germany; 2011 model) for 30 min prior to task-orientated training. Each subject was seated on an armless chair in front of the platform and instructed to flex both shoulders at 90°, slightly bend both elbows, and then bend the trunk forward to allow both hands to be placed on the platform. Each subject was allowed to hold the palms slightly off the platform to minimise discomfort and prevent strong stimulation of the organs, eyes, and head. The WBV protocol featured seven elements at 4 to 7, 8 to 11, 12 to 15, 16 to 19, 12 to 15, 8 to 11, and 4 to 7 Hz. Each element was delivered for 2 min, and 2 min of rest separated the elements. The frequency of each element was increased by 1 Hz weekly. Thus, the frequencies delivered in week 1 were 4, 8, 12, 16, 12, 8, and 4 Hz; those in week 2 were 5, 9, 13, 17, 13, 9, and 5 Hz; those in week 3 were 6, 10, 14, 18, 14, 10, and 6 Hz; and those in week 4 were 7, 11, 15, 19, 15, 11, and 7 Hz to prevent adaptation. The ULC group received ULC training for 30 min before task-oriented training. The intensities (five levels were possible) of both cycles were chosen by the patient. All subjects in both groups received task-orientated training for 30 min after WBV or ULC, including eating (use of a spoon and cup), dressing (donning and removing a shirt), and personal hygiene (use of a toothbrush, comb, and towel). All subjects participated for 60 min/day, 5 days per week, for 4 weeks. All also received conventional physical therapy.

### 2.3. Outcome Measurements

All subjects were assessed at baseline and after intervention. Motor function was measured using the MFT, and grip strength was measured using a Jamar hydraulic hand dynamometer.

The MFT was developed to assess the impairments in motor function of the affected upper extremity of stroke patients and to statistically analyse the possible recovery processes during rehabilitation. The MFT is composed of 32 test items, which examine arm motions and manipulative activities. The test-retest reliability coefficient and interrater reliability of the MFT were consistently above 0.95. Cronbach's *α* coefficient as internal consistency of eight items was also 0.95. With respect to the validity of the MFT, it had a correlation of >0.8 with both the Brunnstrom stage and the Stroke Impairment Assessment Set [22].

Grip strength is useful in clinical practice for the assessment of disease and/or rehabilitation progression. The Jamar hydraulic hand dynamometer was used to measure muscle strength (isometric grip strength test). The participant was asked to squeeze the dynamometer as hard as possible with each of his or her hands. Both maximal handgrip force and endurance were assessed. The Jamar dynamometer was found to be highly reliable = 0.98 and valid = 0.99 for measuring hand grip strength [23].

### 2.4. Statistical Analysis

All data were analysed using the Statistical Package for the Social Sciences (SPSS) version 12.0 for Windows (SPSS, Chicago, IL, USA). The WBV and ULC groups were compared employing the *χ* ^2^ test, the Mann–Whitney *U-*test, or the independent *t*-test. Parameter changes in each group after treatment were compared with the aid of the Wilcoxon signed-rank test, and differences in the changes between the WBV and ULC groups were compared using the Mann–Whitney test. A *p* value < 0.05 was considered to reflect a statistical significance.

## 3. Results and Discussion

### 3.1. Results

The general characteristics of the WBV and ULC groups are shown in Table 1. There was no significant between-group difference in age, sex, type of stroke, days from stroke onset, Mini-Mental Status Examination-Korean version (MMSE-K) or modified Ashworth scale (MAS) scores, or Brunnstrom stage. Also, neither the MFT score (*p* = 0.22) nor the grip strength (*p* = 0.57) differed between the WBC and ULC group preintervention.

After intervention, both groups exhibited significant increases in MFT scores (*p* = 0.001, 0.002, respectively) and grip strength (*p* = 0.001, 0.001, respectively) compared to the preintervention values (Table 2). Furthermore, the MFT score (*p* = 0.016) and grip strength (*p* = 0.023) improved more in the WBV than in the ULC group.

## 4. Discussion

In this study, we found that the use of WBV on motor function and grip strength in patients with subacute stroke was more effective than the use of ULC. Of course, motor function and grip strength improved in both groups, but it improved more in the WBV group. Therefore, task-oriented training after WBV effectively improved motor function and increased grip strength.

All participants had subacute strokes. Although some recovery may occur spontaneously within the first 3 months after stroke [24], intensive training is essential to improve motor recovery [25]. Active upper extremity movement enhances neuroplasticity [26], improving motor recovery [27]. Early active movements were associated with improvements in both groups.

All participants received task-oriented training, which reduces upper extremity impairment and improves both motor function [28] and individual perceptions of health-related quality of life [29]. Misbah and Muhammad (2017) reported that task-oriented training greatly improved upper extremity function in subacute stroke patients [30].

In the previous studies, WBV effectively improved motor function in stroke patients [31, 32]. WBV induced tonic vibration reflexes affected the proprioceptive systems of primary and secondary afferent fibers [18]. The WBV with frequency < 20 Hz induced muscular relaxation [19].

Our study had several limitations. First, the sample size was small, and the results thus cannot be generalised. Second, we did not schedule follow-up after interventions ended; long-term outcomes were not explored.

## 5. Conclusions

These findings suggest that the use of WBV and ULC was effective as remedial treatments for improving upper extremity motor function and increasing grip strength for patients with subacute strokes. The improvement was more pronounced for the WBV treatment.

## Figures and Tables

**Table 1:** Characteristics of participants.

Variables	WBV group (*n* = 30)	ULC group (*n* = 30)	Between-group *p* values
Age (years), mean ± SD	58.7 ± 7.1	60.7 ± 5.9	0.37^1^
Gender			
Male	18	17	0.71^2^
Female	12	16	
Stroke			
Intracranial hemorrhage	15	16	0.46^2^
Cerebral infarction	15	14	
Time since onset of stroke (days), mean ± SD	95.7 ± 31.5	77.3 ± 30.3	0.74^1^
MMSE-K, mean ± SD	27.2 ± 1.32	27.8 ± 1.66	0.31^3^
MAS, mean ± SD	1.00 ± 0.38	1.20 ± 0.56	0.39^3^
Brunnstrom stage, mean ± SD	2.53 ± 0.52	2.40 ± 0.51	0.54^3^
MFT score, mean ± SD	10.93 ± 4.15	12.40 ± 4.48	0.22^3^
Grip strength (kg), mean ± SD	2.36 ± 2.29	2.92 ± 1.95	0.57^1^

SD: standard deviation, WBV: whole-body vibration, ULC: upper and lower cycle, MMSE-K: Mini-Mental Status Examination-Korean version, MAS: modified Ashworth scale, MFT: manual function test, *p* < 0.05. ^1^Independent *t*-test, ^2^ *χ* ^2^ test, and ^3^Mann–Whitney *U-*test.

**Table 2:** Parameters before and after treatment.

	WBV group (mean ± SD)			ULC group (mean ± SD)			Between-group *p* values
	Before treatment	After treatment	*p* value	Before treatment	After treatment	*p* value	
MFT score	10.93 ± 4.15	17.60 ± 5.54	0.001^∗^	12.40 ± 4.48	16.13 ± 5.49	0.002^∗^	0.016^†^
Grip strength (kg)	2.36 ± 2.29	4.40 ± 1.39	0.001^∗^	2.92 ± 1.95	4.09 ± 1.55	0.001^∗^	0.023^†^

SD: standard deviation, WBV: whole-body vibration, ULC: upper and lower cycle, MFT: manual function test, ^∗^ *p* < 0.05 by Wilcoxon's signed-rank test, ^†^ *p* < 0.05 by the Mann–Whitney *U*-test.

## Data Availability

(1) The data used to support the findings of this study were supplied under license and so cannot be made freely available. Requests for access to these data should be made to mubul@kku.ac.kr. (2) The data used to support the findings of this study are currently under embargo while the research findings are commercialized. Requests for data, 12 months after publication of this article, will be considered by the corresponding author. (3) The data used to support the findings of this study may be released upon application to Dr. Kim, who can be contacted at mubul@kku.ac.kr.
